# Reinforced Epithelial Barrier Integrity via Matriptase Induction with Sphingosine-1-Phosphate Did Not Result in Disturbances in Physiological Redox Status

**DOI:** 10.1155/2016/9674272

**Published:** 2015-12-28

**Authors:** E. Pászti-Gere, Á. Jerzsele, P. Balla, G. Ujhelyi, A. Székács

**Affiliations:** ^1^Department of Pharmacology and Toxicology, Faculty of Veterinary Science, Szent István University, István u. 2, Budapest 1078, Hungary; ^2^1st Department of Pathology and Experimental Cancer Research, Semmelweis University, Üllői ut 26, Budapest 1085, Hungary; ^3^Faculty of Pharmacy, Semmelweis University, Hőgyes u. 7, Budapest 1092, Hungary; ^4^Agro-Environmental Research Institute, National Agricultural Research and Innovation Centre, Herman O. u. 15, Budapest 1022, Hungary

## Abstract

*Objectives.* The relationship among matriptase function, cellular redox status, and maintenance of intestinal barrier integrity has not been established yet. The aim of this study is to reveal if the crosstalk between matriptase activators and intestinal epithelial monolayers can lead to perturbations in physiological redox regulation *in vitro*. *Methods.* The effects of suramin and sphingosine-1-phosphate (S1P) were tested on viability of intestinal porcine epithelial IPEC-J2 cells using MTS assay. Measurements of transepithelial electrical resistance (TER) were performed to determine changes in barrier integrity of cell monolayers. Amplex Red assay was used to monitor extracellular hydrogen peroxide production. Occludin distribution pattern was detected prior to and after matriptase activation using immunofluorescent staining technique. *Results.* TER reduction was observed in suramin-treated IPEC-J2 cell monolayers, which could be attributed to cell cytotoxic properties of 48 hr 50 *μ*M suramin administration. In contrast, S1P treatment increased TER significantly and elevated occludin accumulation in tight junctions. It was also found that extracellular hydrogen peroxide levels were maintained in IPEC-J2 cells exposed to matriptase activators. *Discussion.* S1P administration not accompanied by redox imbalance might be one of the key strategies in the improvement of barrier function and consequently in the therapy of intestinal inflammations.

## 1. Introduction

Intestinal epithelium provides strong barrier against noxious chemicals and enteropathogens. Several studies were conducted on nontumorigenic neonatal porcine small intestinal epithelial IPEC-J2 cells on microporous membranes to assess resemblance of this cell line to monolayer epithelium* in vitro* [1, 2] and to determine the effects of oxidative stress and bacterial, fungal infections on barrier integrity [3–8]. IPEC-J2 cells become polarized after formation of apical junctional complex and the rate of functional integrity can be measured via development of transepithelial electrical resistance (TER). They behave similarly to human colon adenocarcinoma cells (Caco-2 and T84 cells) with the advantage of not being cancerous, and their glycosylation pattern, proliferation rate, and colonisation ability are closer to physiological functioning of enterocytes [9].

Cell surface proteolysis is an important process in development and maintenance of healthy epithelial homeostasis via proper functioning of type II transmembrane serine protease, matriptase. The regulation of intestinal barrier integrity via matriptase modulation is one of the key pillars in the normal gut physiology. If the epithelial layer becomes inflamed due to loss of matriptase activity, increased paracellular permeability and lower TERs could be detected [10]. It was proven previously by us that selective inhibition of matriptase with 3-amidinophenylalanine-derived MI-432 weakened significantly the epithelial monolayer barrier function, thus showing indirectly that matriptase takes part in membrane dynamics and partial loss of matriptase activity could affect negatively the intestinal epithelial barrier competence [11]. It was also found that imbalance in redox status could deteriorate epithelial barrier integrity via multifaceted modes of actions including altered distribution pattern of transmembrane trypsin-like serine protease activity [12].

Cellular events responsible for autoproteolytic matriptase activation include oligomerization of matriptase zymogens and hepatocyte growth factor activator inhibitor (HAI-1) and conversion of single-chain zymogen to two-chain active protease. After activation matriptase-HAI-1 complex is shed into the extracellular milieu. Two matriptase activation inducers such as lysophospholipid-derivative, sphingosine 1-phosphate (S1P), and polyanionic compound, suramin, were found to act cell-type specifically [13].

S1P is an active lipid generated by hydrolysis of glycerophospholipids and sphingomyelin in the membranes of activated cells including kinase-mediated phosphorylation of sphingosine. It was reported that S1P released from activated platelets produces elevated transmonolayer electrical resistance as an indicator of significant endothelial cell barrier enhancement in human pulmonary artery endothelial cells, HPAEC, which was accompanied by increased cortical actin and rapid translocation of cortactin to the cell periphery [14, 15]. Matriptase, its exogenous activation inducers and HAI-1 could accumulate at activation foci thus ensuring well-organized switched on-off mechanisms of matriptase-mediated proteolysis in human immortalized epithelial cells, 184 A1N4 [16].

The aim of this study was to investigate the effects of matriptase activation on intestinal epithelial integrity in porcine nontumorigenic nonpolarized and differentiated IPEC-J2 cells cultured on membrane insert after estimation of cell cytotoxic properties of the applied matriptase activators, S1P and suramin. It was also tested if changes in TERs can be attributed to alterations in extracellular hydrogen peroxide levels detected with Amplex Red fluorescence method. In addition, immunofluorescence staining of occludin was used to determine if link exists between exogenously induced matriptase activation and localization pattern of tight junctional occludin.

## 2. Materials and Methods

### 2.1. Cell Lines and Culture Conditions

The IPEC-J2 cell line used in this study was derived from jejunal epithelia of a neonatal piglet. It is a nontransformed cell line that in some respects mimics* in vivo* conditions when cultured on membrane inserts. Cells form a differentiated layer and are attached to each other via tight junctions apically. IPEC-J2 cells were seeded at a density of 1.5 × 10^5^ per well on six-well plates with Transwell polyester membrane inserts (pore size 0.4 *μ*m; surface area 4.67 cm^2^; Sigma, Germany) coated with rat tail collagen (Sigma) in a 1.5 mL apical and 2.6 mL basolateral volume. Cells were maintained in complete medium containing 1 : 1 mixture of Dulbecco's Modified Eagle's Medium and Ham's F-12 Nutrient Mixture (DMEM/F12) supplemented with 5% FBS, 5 *μ*g/mL insulin, 5 *μ*g/mL transferrin, 5 ng/mL selenium, 5 ng/mL epidermal growth factor, and 1% penicillin-streptomycin (all from Fisher Scientific, USA). Cell cultures were tested by PCR and were found to be free of mycoplasma contamination. Cells were allowed to adhere for 24 h before being washed and refed every other day until confluence. They were grown at 37°C in a humidified atmosphere of 5% CO_2_.

### 2.2. Exposure of IPEC-J2 Cells to Matriptase Activators

Before treatment nondifferentiated and polarized IPEC-J2 cells were washed twice with plain medium. Matriptase activators, S1P und suramin, were added both apically and basolaterally for different time intervals. After incubation, the cells were washed twice with plain medium before being subjected to the subsequent procedures.

### 2.3. MTS Assay for Cell Viability

Influence of matriptase activators, suramin (at 50, 100, and 200 *μ*M) and sphingosine-1-phosphate (S1P) (at 50, 100, and 200 ng/mL diluted from 0.5 mg/mL stock solution prepared in methanol) in phenol red-free DMEM on the viability of enterocytes was tested. IPEC-J2 cells were seeded in a 96-well plate and incubated with S1P and suramin for up to 48 hr in treated groups. The control cells were incubated only with phenol red-free DMEM. After removal of the medium and washing the cells 3-fold with phosphate-buffered saline buffer (PBS), 20 *μ*L of CellTiter96 aqueous one solution (Promega, Bioscience, Budapest, Hungary) containing tetrazolium compound, MTS, and an electron coupling reagent, phenazine ethosulfate, was pipetted into each well of the 96-well assay plate containing the samples in 100 *μ*L of culture medium. The plate was incubated with dye for 2 hr in a humidified, 5% CO_2_ atmosphere. Viability of IPEC-J2 cells was measured at 490 nm using EZ Read Biochrom 400 microplate reader after 48 hr treatment with matriptase activators.

### 2.4. Transepithelial Electrical Resistance

IPEC-J2 cells were plated to confluence in 6-well polyester membrane inserts. The cells were washed threefold and incubated with 50 *μ*M suramin and 200 ng/mL S1P for different time intervals (for 0.5, 2, 24, and 48 hr) in phenol red-free DMEM. TER measurements of cell layers were performed prior to and after administration of matriptase activators using EVOM Epithelial Tissue Volt/Ohmmeter (World Precision Instruments, Berlin, Germany). The average baseline electrical resistance of the polyester membrane insert without IPEC-J2 cell layer was 113 Ω, which was subtracted from actual TER results.

### 2.5. Extracellular H_2_O_2_ Measurement Using Amplex Red Method

Fluorescence ROS measurement of cell supernatant was based on the detection of H_2_O_2_ using the Amplex Red Hydrogen Peroxide Assay Kit (Invitrogen, Molecular Probes). In the presence of horseradish peroxidase (HRP), Amplex Red reacts with H_2_O_2_ in a 1 : 1 stoichiometry producing a highly fluorescent resorufin [17]. IPEC-J2 cells were treated with suramin at 50 *μ*M or with S1P at 200 ng/mL for 0.5, 2, 24, and 48 hr in phenol red-free DMEM and the H_2_O_2_ concentrations in the medium were determined using the working solution of 100 *μ*M Amplex Red reagent and 0.2 U/mL HRP. After 30 min incubation with the dye at room temperature the quantitative analyses of H_2_O_2_ contents were accomplished, the excitation wavelength was set at 560 nm, and emission was measured at 590 nm (Victor X2 2030 fluorometer).

### 2.6. Investigation of Occludin Distribution via Immunofluorescence Staining

Inserts were fixed in methanol for 5 min followed by bovine serum albumin (BSA) (1%, Sigma Aldrich, St. Luis, MO) protein block for 20 min. Sections were incubated for 1 hr in a humid chamber at room temperature with anti-occludin polyclonal primary antibody (1 : 200, Sigma-Aldrich). For secondary antibody Alexa546 (orange-red) anti-rabbit Ig-s diluted in 1 : 200 was used for 1 hr. Cell nuclei were stained in blue using 4′,6-diamidino-2-phenylindole (DAPI) (1 : 500, Invitrogen-Molecular Probes) for additional 10 min. Between incubations the slides were washed in PBS for 3 × 2 min. Membranes were attached on glass slides using fluorescence mounting medium (DAKO, Glostrup, Denmark). The samples were analysed using Olympus IX73 inverted microscope with 100 W Hg fluorescent accessory (Unicam Magyarország Kft., Budapest, Hungary) with LUCIA Cytogenetics 2.5 software.

### 2.7. Statistical Analysis

For statistical evaluation R 2.11.1 software package (2010) was applied. Differences between means were evaluated by one-way analysis of variance (one-way ANOVA) with* post hoc* Tukey test, where data were of normal distribution and homogeneity of variances was confirmed. Differences were considered significant if the *p* value was <0.05.

## 3. Results

### 3.1. Assessment of Cell Viability after Matriptase Activation

**Figure 1:**
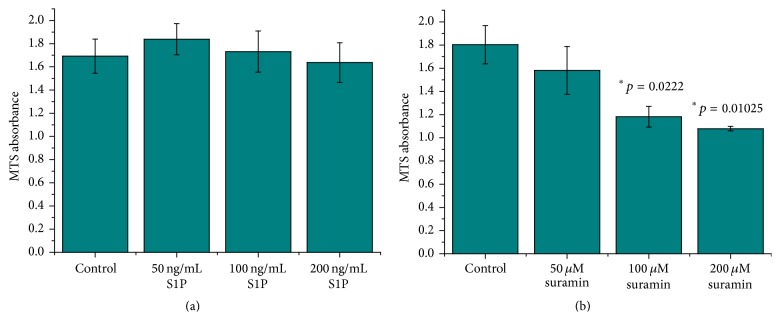
(a) 48 hr incubation of IPEC-J2 cells with S1P at different concentrations at 50, 100, and 200 ng/mL. Values represent average absorbance values of produced MTS formazan in metabolically active cells ± SEMs. No significant differences were found between control and cell monolayers exposed to S1P at concentration up to 200 ng/mL. (b) 48 hr incubation of IPEC-J2 cells with suramin at different concentrations at 50, 100, and 200 *μ*M. Values represent average absorbance values of produced MTS formazan in metabolically active cells ± SEMs. Significant differences were found between controls and cell monolayers treated with higher concentrations (100 *μ*M, ^*∗*^ *p* = 0.0222 and 200 *μ*M, ^*∗*^ *p* = 0.01025) of suramin.

Cell cytotoxicity assays were performed to evaluate the highest concentration of S1P and suramin, which did not cause significant cell death. It was found that S1P at up to 200 ng/mL concentration can be used safely for 48 hr in IPEC-J2 cells, and thus this concentration could be applied in further experiments to determine potential barrier protective effect of S1P via matriptase modulation (Figure 1(a)). However, suramin at 100 and 200 *μ*M concentrations decreased cell survival to great extent indicating that only 50 *μ*M suramin for 48 hr can be administered to the cell monolayers to see the effect of matriptase activation on barrier integrity without massive loss of IPEC-J2 cells (Figure 1(b)).

### 3.2. TER Measurements of IPEC-J2 Monolayers

**Figure 2:**
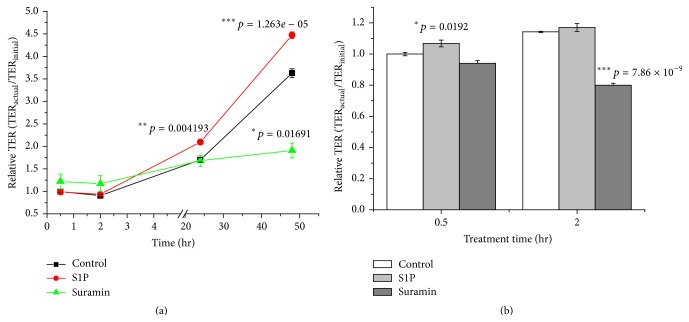
(a) Transepithelial electrical resistance values of IPEC-J2 cell monolayers after 0.5, 2, 24, and 48 hr treatment with S1P (200 ng/mL) and suramin (50 *μ*M) were measured and compared to controls in nine parallel experiments. At the start of the treatment IPEC-J2 cells were not completely differentiated. The values are expressed in average relative TERs ± SEMs. Asterisk indicates significant differences between treated and mock groups (^*∗*^ *p* < 0.05, ^*∗∗*^ *p* < 0.01, and ^*∗∗∗*^ *p* < 0.001). (b) Transepithelial electrical resistance values of differentiated IPEC-J2 cell monolayers after 0.5, 2 hr treatment with S1P (200 ng/mL) and suramin (50 *μ*M) were measured and compared to controls in nine parallel experiments. The values are expressed in average relative TERs ± SEMs. Asterisk indicates significant differences between treated and mock groups (^*∗*^ *p* < 0.05 and ^*∗∗∗*^ *p* < 0.001).

Changes in barrier integrity were assessed via measurements of TERs between apical and basolateral compartments of control and treated IPEC-J2 cell monolayers exposed to S1P (200 ng/mL) and to suramin (50 *μ*M) for 0.5, 2, 24, and 48 hr. In case of S1P, TERs showed significant changes compared to controls when treatment was continued for 24 (*p* = 0.004193) and 48 hr (*p* = 1.263 × 10^−5^) (Figure 2(a)). Increases in TERs in IPEC-J2 cells treated with the lowest S1P concentration (50 ng/mL) could not be observed even after 48 hr S1P administration (*data not shown*). It was found that 48 hr treatment of IPEC-J2 cells with suramin decreased TER significantly (*p* = 0.01691) (Figure 2(a)).

To elucidate the effects of matriptase activators on TERs of differentiated IPEC-J2 cells suramin (50 *μ*M) and S1P (200 ng/mL) were administered for 0.5 and 2 hr. It was found that S1P exerted barrier protective effect after 0.5 hr S1P addition (*p* = 0.0192) on contrast to suramin, which deteriorates barrier integrity by lowering significantly TERs of IPEC-J2 cell monolayers (*p* = 7.86 × 10^−9^) after 2 hr treatment (Figure 2(b)).

### 3.3. Measurement of H_2_O_2_ Production

**Figure 3:**
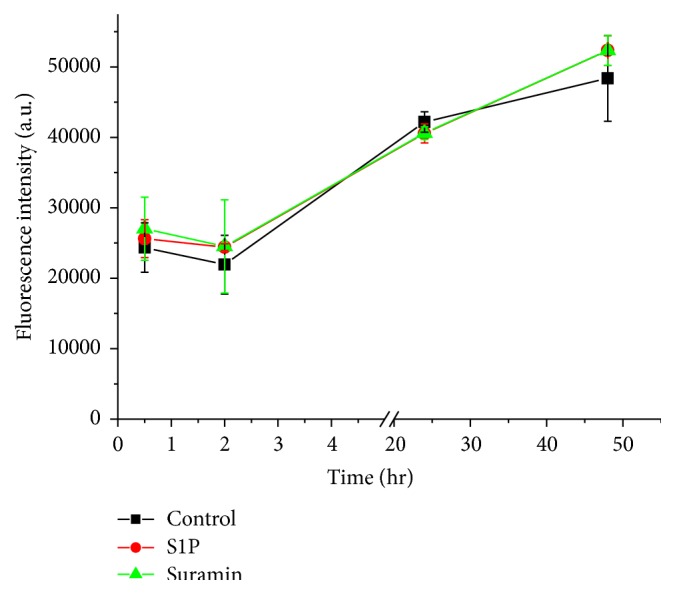
Fluorescence intensities of supernatants of control and S1P (200 ng/mL)- and suramin (50 *μ*M)-treated groups determined with Amplex Red assay. At the start of the treatment IPEC-J2 cells were not completely differentiated. The measured values are indicated as average fluorescence intensities ± SEMs (*n* = 5). There were no significant differences between mock and matriptase activator-treated groups (*p* > 0.05) up to 48 hr.

Amplex Red assay was used to detect extracellular hydrogen peroxide production after matriptase activation. Suramin (50 *μ*M) or S1P (200 ng/mL) was added for 0.5, 2, 24, and 48 hr apically to IPEC-J2 cell monolayer and supernatants were analysed by fluorimeter. It was established that neither suramin nor S1P could generate higher extracellular peroxide amounts. It was also found that barrier dysfunction observed when cell monolayers were exposed to suramin for 48 hr could not be attributed to excessive extracellular H_2_O_2_. The increases in fluorescence signals can be explained that during cell growth the baseline H_2_O_2_ signal was elevated both in controls and in IPEC-J2 cells with enhanced matriptase activity (Figure 3). In case of differentiated IPEC-J2 cells 0.5 and 2 hr treatment with S1P and suramin did not result in significant increases in fluorescence signal intensities showing that extracellular H_2_O_2_ production was not altered during matriptase activation and changes in TERs could not be attributed to the elevated amount of extracellular peroxide contents. In controls the average fluorescence intensity ± SEM was 23749 ± 1338 versus 25830 ± 157 measured extracellularly in the medium of S1P-treated IPEC-J2 cells after 30 min incubation. Amplex Red fluorescence intensities did not differ in extracellular milieu of control (20370 ± 654, *p* = 0.198) and suramin-administered (21512 ± 1521, *p* = 0.528) IPEC-J2 cells significantly after 2 hr treatment.

### 3.4. Occludin Localization Pattern in S1P and Suramin-Treated Intestinal Epithelial Cells

**Figure 4:**
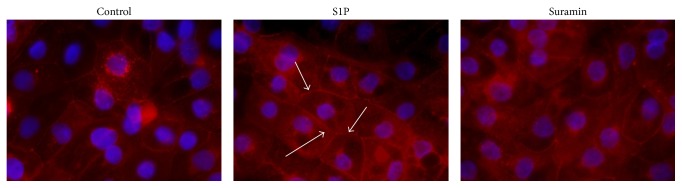
Immunofluorescence staining of occludin (in red) in control and in S1P (200 ng/mL) and suramin (50 *μ*M)-treated IPEC-J2 cells after 48 hr treatment. Occludin can be seen in red and cell nuclei were stained with DAPI in blue. S1P-induced more intensive occludin staining in cell membranes, which was indicated by white arrows. In case of suramin administration, occludin shows more diffuse distribution pattern compared to that in control panel. 600x magnification.

Occludin localization was studied in untreated control and in matriptase activator-treated differentiated IPEC-J2 cells using immunofluorescence staining method. The cells were investigated 48 hr after S1P and suramin treatment. In controls, occludins localized at cell-cell junctions of polarized monolayers of IPEC-J2 cells. In IPEC-J2 cells exposed to S1P at 200 ng/mL presence of occludin in junctional protein assembly was enhanced; thus higher rate of occludin accumulation was observed in TJ strands to that detected in control groups based on more intensive immunofluorescence staining pattern. Suramin at 50 *μ*M concentration, however, resulted in more diffuse staining distribution of occludin (Figure 4).

## 4. Discussion

Type II transmembrane serine protease, matriptase, was identified as a critical component of preserved integrity of epithelial barrier function; moreover, its function has been evidenced to be inhibited by low molecular weight dipeptide amides and is expected also to be affected by other zwitterionic amino acid derivatives, as well as by membrane disrupting surfactants (e.g., tallow amine derivatives).

Downregulated matriptase and consequent loss of matriptase activity in inflammatory bowel diseases led to persistent dextran sodium sulfate-induced colitis and prolonged, life-threatening inflammation in* St14* hypomorphic mice [18]. The siRNA silencing of matriptase expression or inhibition of matriptase activity disrupted development of epithelial barrier in Caco-2 cells, which resulted in significant decrease in TERs of cell monolayers and increased paracellular permeability to macromolecules such as 4 kDa fluorescein isothiocyanate (FD4)-labelled dextran [10]. We first proved that selective inhibition of matriptase with 3-amidinophenylalanine-derived MI-432 contributed to dramatic decrease in TERs indicating barrier dysfunction and impaired cell monolayer [11].

These findings raise the question whether induction of matriptase activation can enhance barrier formation, thus alleviating severity of bowel inflammation in pathological conditions such as Crohn's disease and ulcerative colitis. It was noted that matriptase supplementation could prevent partially inflammatory mediators-associated disruption of tight junction responsible for modulation of barrier function, but treatment was less efficient when canine epithelial cell line, SCBN, was exposed to higher doses cytokines [19, 20].

Matriptase activation can be an outcome of a cellular response to pH decrease via perturbation of intracellular pH homeostasis by application of amiloride-derivatives or diisothiocyanostilbene-2,2′-disulfonic acid. Acidosis-driven matriptase activation in epithelial cells enables protein-protein interactions leading to activational cleavage of matriptase in a very short time [21]. In addition to autoactivation processes triggered by pH changes in extracellular milieu, exogenously added inducers such as S1P, androgen and suramin can also activate matriptase pools. Treatment of 184A1N4 and MCF 10A human mammary epithelial cells with serum resulted in activation of matriptase [22]. It was also observed that structurally related compounds such as dihydro-S1P, ceramide 1-phosphates can also activate matriptase, however, only at significantly higher concentration [23]. S1P seems to be an important enhancer of cellular barrier function of the vascular endothelium, and it can induce cytoskeletal rearrangement and elevated transmonolayer electrical resistance in pulmonary artery endothelial cultures. Intravenously administered S1P at 1 *μ*M is also capable of attenuation of vascular leakage in murine and canine models of acute lung injury [24].

Despite the growing body of evidence that S1P plays crucial role in the enhancement of endothelial barrier integrity and in spite of the fact that S1P and its receptors are highly expressed in intestinal tissues only few studies have been published recently focusing on the relationship between matriptase induction and intestinal epithelial barrier reinforcement. The levels of adherens junctional E-cadherin protein and mRNA were investigated in differentiated intestinal epithelial cells, IEC-6, upon S1P treatment. It was proven that E-cadherin localized at cell-cell contact in higher amounts leading to cytoskeletal rearrangements and S1P could decrease ^14^C mannitol paracellular permeability contributing to improved barrier integrity under physiological and pathological conditions [25]. It was also ascertained that abundance in some tight junctional proteins was observed in intestinal epithelial cells with upregulated expression of sphingosine kinase 1 (SphK1) [26]. Until now there was no any report in which intestinal epithelial cells were exposed to suramin to study the effect of chemical inducer of matriptase on intestinal barrier integrity.

Based on our results TERs of monolayers increased significantly when IPEC-J2 cells were treated with S1P at 200 ng/mL for 24 and 48 hr. Our experiments were also conducted with the same concentration of inducer in polarized IPEC-J2 cells but for shorter duration of time and it was confirmed that 0.5 hr administration of S1P improved barrier integrity to great extent. These data were in accordance with other study which reported that S1P administration significantly improved barrier integrity via modulating expression and cortical localization of E-cadherin, which led to decreased permeability and concomitant elevation in TER in rat intestinal epithelial cells [25]. We also investigated if matriptase activation could cause tipped redox balance between reactive oxygen species and physiologically occurring antioxidants. It was ascertained that independently of polarization degree of the cells the extracellular hydrogen peroxide production was not elevated or was compensated efficiently by redox mechanisms in IPEC-J2 exposed to S1P. Thus, treatment with S1P could offer a new strategy to reduce occurrence of leaky gut syndrome accompanied by enhanced paracellular permeability or to restore inflamed intestinal epithelium without the risk of cytotoxic side effects and without introduction of excessive oxidative stress. Suramin, another matriptase activator, could not strengthen barrier integrity and the lack of its protective effects resulted in remarkable decrease in TER in polarized IPEC-J2 cells exposed to 48 hr suramin treatment. Completely differentiated IPEC-J2 cells responded more sensitively to 50 *μ*M suramin administration since 2 hr incubation deteriorated monolayer integrity detected as significant TER reduction. Interestingly, it was also noted that there are differences between S1P-induced matriptase translocation to cell-cell junctions, which is F-actin polymerization-dependent process and F-actin polymerization-independent activation at vesicle-like structures induced by suramin application in the human mammary epithelial cells [16].

The elucidation of paracellular gate modulation can represent useful contribution toward understanding of altered mechanisms of inflamed intestinal epithelium under pathological conditions. Tight junctions contain several types of proteins such as claudins, occludin, and junctional adhesion molecules. In-depth assessment of the impact of junctional proteins on the reinforcement of epithelial monolayer integrity is of key importance in early prevention of barrier dysfunction. Many studies have shown that occludin takes part in the maintenance of gate function of tight junction in different epithelial cell models. It was shown that overexpression of occludin in Madin-Darby canine kidney cells caused significant increase in steady-state TER [27] and on the other side, murine epithelial cells CSG 120/7 equipped with mutant N-terminally truncated occludin showed decreased TER and elevated paracellular FD4 flux; thus this latter cell monolayer failed to show efficient barrier function [28]. It was also reported that occludin siRNA transfection caused enhanced flux of various probes such as inulin and dextran across Caco-2 cell monolayers [29]. In accordance, we first report here that in nontumorigenic intestinal IPEC-J2 cells occludin immunofluorescence staining became more intense after 48 hr S1P-mediated matriptase activation compared to that detected in mock cells. Thus, reinforcement of intestinal epithelial barrier integrity expressed in increase rate in TERs can be explained via occludin accumulation at cell-cell junctions. In contrast, suramin-treated IPEC-J2 cells did not show favourable TER alteration and occludin staining pattern, indicating that suramin treatment did not exert protective effect on monolayer barrier function.

## 5. Conclusion

Taken together, it has been shown that matriptase activation with S1P at 200 ng/mL increased the intestinal epithelial barrier integrity. This was confirmed by detection of both significant enhancement of TERs of IPEC-J2 cell monolayers and occludin accumulation at cell-cell contacts. Usage of S1P did not appear to perturbate cellular redox status based on our* in vitro* findings. The lack of the adverse effect such as excessive oxidative stress when S1P is applied in IPEC-J2 cells raises the possibility that S1P might act as a safe barrier integrity enhancer capable of counteracting the enhanced paracellular permeability. Thus, application of S1P seems to be an effective approach to strengthen barrier function and to exert its beneficial effect in the prevention or in the treatment of intestinal inflammatory diseases.
